# Mobility and Active Ageing in Suburban Environments: Findings from In-Depth Interviews and Person-Based GPS Tracking

**DOI:** 10.1155/2012/257186

**Published:** 2012-12-17

**Authors:** Elisabeth Zeitler, Laurie Buys, Rosemary Aird, Evonne Miller

**Affiliations:** School of Design, Faculty of Creative Industries, Queensland University of Technology, Gardens Point Campus, 2 George Street, GPO Box 2434, Brisbane, QLD 4000, Australia

## Abstract

*Background*. Governments face a significant challenge to ensure that community environments meet the mobility needs of an ageing population. Therefore, it is critical to investigate the effect of suburban environments on the choice of transportation and its relation to participation and active ageing. *Objective*. This research explores if and how suburban environments impact older people's mobility and their use of different modes of transport. *Methods*. Data derived from GPS tracking, travel diaries, brief questionnaires, and semistructured interviews were gathered from thirteen people aged from 56 to 87 years, living in low-density suburban environments in Brisbane, Australia. *Results*. The suburban environment influenced the choice of transportation and out-of-home mobility. Both walkability and public transportation (access and usability) impact older people's transportation choices. Impracticality of active and public transportation within suburban environments creates car dependency in older age. *Conclusion*. Suburban environments often create barriers to mobility, which impedes older people's engagement in their wider community and ability to actively age in place. Further research is needed to develop approaches towards age-friendly suburban environments which will encourage older people to remain active and engaged in older age.

## 1. Introduction

Population ageing is a global phenomenon. By 2051, it is estimated that 28% of the Australian population will be aged 65 years and older, representing a doubling of this older cohort from 2004 [1]. Most of Australia's ageing population (64%) reside in urban areas [2], characterised by a predominance of low-density suburban environments [3]. As most older Australians intend to “age in place” and remain living in their community as they get older [4], urban planners and policymakers are focused on ensuring that the design of the urban environment meets their changing needs. Issues of health, housing, income, and mobility typically dominate policy discussions, with increasing acknowledgment that the quality of life for older people depends on them being able to maintain their participation within the community in preferred out-of-home activities [5]. Thus, this paper specifically investigates if and how characteristics of suburban environments might impact older people's mobility, transport-mode choices, and participation in community activities. 

Mobility, succinctly defined as “the fundamental physical capacity to move” [6, page 782], is important for active ageing. The World Health Organization's [7] definition of active ageing identifies participation (alongside health and security) as one of three key contributors to quality of life in older age. Healthy and active living in older age is conceptualised as an outcome of an ageing process that allows equal opportunities and treatment for people at all stages of their life, regardless of their personal characteristics [7, 8]. The community environment should enable everyone to stay active and engaged, regardless of varying needs and capacities. The built environment, which describes all aspects of the environment created and built by humans, has a central role in facilitating older people's opportunities for health, participation, and security [9]. Critically mobility is often central to enabling older people's participation—particularly when they reside in suburban communities where the characteristics of the built environment and transport infrastructure may either enable or impede their participation in out-of-home activities. 

Research has demonstrated that the built environment has an influence on quality of life and mobility, as it facilitates safe, accessible, and affordable services in reasonable travel time [10]. Neighbourhood characteristics such as affluence, better amenities, and facilities also promote higher levels of social activity, although social contact is unrelated [11]. The walkability of neighbourhoods is critical to active ageing, as it inhibits or allows the integration of physical activity into daily routines and fosters interaction with others [12]. Specific neighbourhood characteristics (such as density, greater number of safe street intersections, and green and open spaces) have been found to positively influence walking activity in older age [13]. The use of public transport options is generally found to be difficult in older age because of service design and provision, vehicle accessibility, provision of information, other people, and personal mobility [14]. Older drivers also change the use of their car, due to factors such as retirement, age, and health as well as difficult traffic situations [15] and might therefore also face reduced mobility. However, while there is evidence that the environment sets the context for out-of-home mobility, it remains unclear to what extent the built environment impacts the use of different transport options in older age [16] and thus its effects on older people's capacity for active ageing.

The idea, that community environment affects out-of-home mobility in older age, is based on the assumption that the interplay between the individual's competences and environmental characteristics determines the optimal functioning of the individual [17, 18]. The holistic approach to mobility taken by Webber et al. [19] demonstrates the complex relationship between between individual determinants (such as cognitive, physical, psychosocial, and financial), cultural determinants (gender, culture and biographical influences), and environment determinants of mobility. This mobility model illustrates that diverse life-space environments (such as home, outdoors, neighbourhood, etc.) involve the interaction of mobility determinants at different levels. A number of cross-national European studies have investigated the complex mobility issues of older people in relation to either their urban or rural environment [20, 21]. One study focused on older peoples day to day mobility and the complex interplay of personal resources and the physical and social environment [20]. It was found that health, housing, the environment, and social network resources impact older people's out-of-home mobility [20]. Further, the use of transport options differs between rural and urban environments and between nations. While walking is used most, especially in combination with services, public transportation is used little in rural settings and more in urban settings. Familiarity with an area positively influences out-of-home mobility [20]. Another study focused on the perspective of older people and experts on the current mobility situation in older age [21]. Mobility was found to be critical to fundamental needs of daily life, walking, and leisure activities. Mobility is also important to maintain a positive self-perception, especially when it allows participation in caring activities (childcare, care for other people), with being mobile also adding to quality of life itself [21].

In America, over half of older people live in highly car-dependent suburban environments [22], which is mainly related to the non-availability of alternative transport options and impractical walking conditions [23]. Suburban environments create mobility constraints and difficulties in older age and adaption processes of mobility practices were found to be important to avoid having to move somewhere else [24]. The reality is that driving is essential within suburban environments for active daily life and allowing independent and autonomous living [25]. In order to stay in their suburban environment, older people change their daily routines (unconsciously) and with this their travel behaviour on an ongoing basis [26]. This is critical as these adaption strategies often result in the reduction or fragmentation of space used within the community, which might lead to a significant loss of autonomy [25]. 

As the majority of older Australians live in suburban environments, it is critical for policy makers and urban planners to identify strategies to enhance out-of-home mobility in those environments. While the literature identifies the built environment as an influence on travel behaviour, the direct effect of suburban environments on older people's travel behaviour, and consequently their mobility remains rather unclear. Thus, this current study explores how low-density suburban environments impact the use of different transport options in older age and discusses its consequences for active ageing.

## 2. Methods

This research uses a qualitative research design. Investigating the link between active ageing, mobility, transport options, and the built environment is a complex undertaking. Therefore, a range of instruments was used to collect data for an in-depth analysis exploring the effect of the community environment on older people's travel behaviour. This paper focuses specifically on mobility aspects, with the data for the cases (*n* = 13) including sociodemographic characteristics, residential location and character, available travel options, preferred or non-preferred built environment, and real-time measurement of travel behaviour. Ethical approval for this research was given by the Queensland University of Technology (QUT) Human Research Ethics Committee.

### 2.1. Sample

This study focuses on the experiences and travel behaviour of older Australians residing in 11 different low-density suburbs across Brisbane (the state capital of Queensland), with a range of 5.05 to 27.74 people per hectare [27]. These suburbs are typified as residential areas with lone standing family homes with yards, and pockets of business areas with shopping centres or strips with shops and facilities. The data used for this study were collected as part of a larger project exploring active ageing and liveability in rural, regional, suburban, and urban locations. This research focuses on data concerning older people residing in low-density suburban environments. Industry partners cooperated by recruiting participants aged 55 years and older. As one purpose of the study was to investigate the differing perceptions and experiences of older people of various ages, the age of the participants for this study ranges from 57 to 87 years.  Table 1 illustrates participants sociodemographic characteristics, with the majority retired (only one was still working part time) and females on average were 7.75 years younger than males.

### 2.2. Data Collection

**Figure 1:**
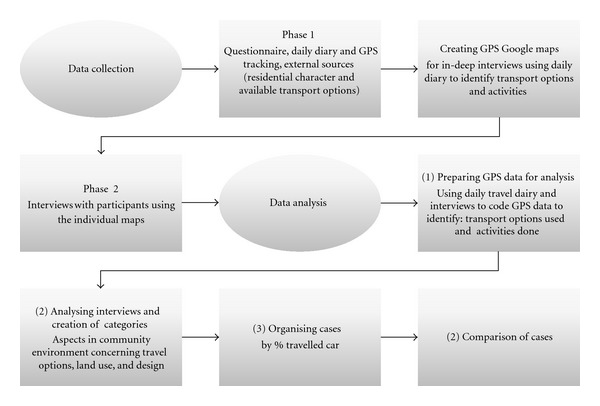
Data collection and analysis.

Participants' data were gathered in 2010 in two phases (see Figure 1), as part of a larger project with a focus on ageing and liveability in rural, regional and urban locations. 

**Figure 2:**
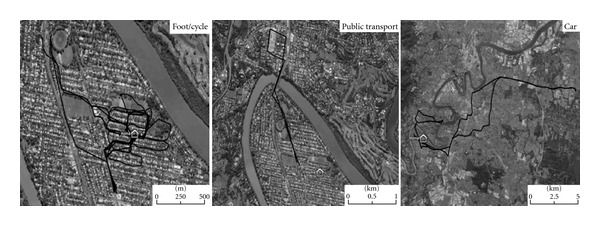
Maps showing use of transportation by one participant.

Firstly, a travel diary (including a brief questionnaire) was handed out in combination with a GPS tracking device [28], to collect data on travel behaviour and out-of-home activities, over seven consecutive days. Secondly, semi-structured interviews were conducted. Those were aided by individual Google Earth maps [29], showing activities and transport options used during the week of tracking (see Figure 2). The maps were created prior to the interviews by using the travel diaries and the GPS data. The use of transport options was coded in different colours. The main focus of the interviews was to explore how participants conceptualised the liveability of their respective communities. All interviews were audio-recorded. A brief questionnaire in the travel diary assessed key sociodemographic characteristics (see Table 1) and participants' reflections about their activities. External sources were used to identify residential characteristics, such as distance to CBD [30], population density [27], and available transport within a five to seven minutes walk [31]. 

#### 2.2.1. Measures: Residential Location and Character and Available Travel Options

Distance to CBD and public transport was represented in kilometres and population density as people per hectare. Participants were asked during their respective interviews what they would do if they could not drive anymore or if they could not maintain the way they currently move around when travelling outside of home. Five categories were developed from participants' responses to this question, namely: “would need to relocate elsewhere”; “could stay with help from family”; “could stay by changing current transport mode”; “could stay by using local services”; “not thought about.”

#### 2.2.2. Measures: Preferred or Non-Preferred Aspects of the Built Environment

In the questionnaire, participants were asked questions about their local community: why they live here, whether they liked it, and how long they thought they could live there. A thematic analysis of the interview transcripts explored in depth participants' preferred and non-preferred aspects of their community, focussing on built environment characteristics, transport options, land use, and design (see Table 2).

#### 2.2.3. Measures: Travel Behaviour

Data on participants' travel behaviour was collected using GPS tracking and a questionnaire (see Table 3) that asked participants “How do you get around?” (Options: “*I walk*”; “*I use a bicycle*”; “*I drive myself* with a: “car”, “motorcycle”, “motored wheelchair”, “mobility scooter”; “*someone else drives me*”: “my partner,” “my children/grandchildren,” “community members,” “social or senior services”; “*I use public transport*”: “bus”, “train”, “taxi”, “ferry”; “*I would like to use public transport but*”: “It is not available in our community”, “It does not go where I need it to go”; “It is too far away from my home”, “It is too expensive”, “I do not feel safe”, “It is too hard to use”). The maps, the travel diary, and interview responses were also used to code the GPS data. Two main categories emerged: travel by transportation (by car as driver, by car as passenger, bus, train, walking, cycling, and ferry) and out-of-home activities, which were classified as daily life activities (e.g., shopping, health) and social activities (e.g., meeting friends, volunteering). This paper focuses on the data about travel by transportation.

### 2.3. Data Analysis

Researchers assigned each participant a unique code number. Each individual's GPS data were analysed to determine the distance travelled per mode of transport used (in kilometres) and the destinations reached (representing activities). All interview audiotapes were transcribed verbatim. The text of the transcripts was manually coded for relevance to preferred and non-preferred aspects of the built environment and its components (transport options land use, and design). All measures were grouped by participant and the amount of car use for transportation (100% car, 90–99% car, 75–77% car, and 0% car). These groups were compared to each other in order to assess whether demographic characteristics, residential location and character, as well as preferred and non-preferred features of the built environment influence travel behaviour. 

## 3. Results

### 3.1. Demographic Characteristics, Residential Location and Character, and Travel Options

Multiple factors, such as socio-demographic characteristics, residential location and character and available travel options, might influence mobility (see Table 1). In this research, the car was the predominant transport choice; five older people drove by car for all trips made during the monitored week and four used the car for between 90–99% of their transportation. People within these two groups were, on average, older than people who drove 75–77% of the distance, or not at all. Of the thirteen participants, seven were married and one widowed, all eight travelled 90–99% or 100% by car.

The character and location of participants' residential areas varied considerably (see Table 1). Seven participants lived less than ten kilometres and six lived ten to twenty kilometres from the CBD. Most people had access to a car and public transport within a five to seven minute walk. However, the frequency of available transportation options varied widely. People living closer to the CBD tended to have a more frequent bus service than those living further away. While only four participants had access to quarter-hourly services (three of these lived within six kilometres of the CBD), nine participants had access to only half-hourly or less frequent bus services. Services were more frequent during peak hours for three participants in this group. The frequency of services was generally lower for people who drove 100% of kilometres by car. 

Participants' perceptions of the suitability of their environment to allow them to age in their present location also varied across the sample. Out of the nine participants who drove 90–99% or 100% by car, five stated that they would have to relocate if their physical mobility declined. In the whole sample of thirteen participants four said that they would need to change their current transportation mode, while two said they could remain in their current location with the help of family and friends. 

### 3.2. Preferred and Non-Preferred Features of the Built Environment

Features of the built environment, preferred and non-preferred, also have an impact on liveability and travel behaviour (see Table 2). Participants described proximity to family and friends being significant to where they lived: “*But that is an important part of where we live, is having reasonable access to your kids.” (P12). *Proximity to shops and services was also most commonly cited reasons for living within the current environment: “*We always said as we got older we would be going back to the city, where you have got the services.” (P6)*

Most participants said they would like to live in their community as long as they were able to live independently. All those participants who stated in the interview that they would have to move away from their current neighbourhood when their physical mobility declined used the car for 90–99% or 100% of their travel. One participant highlighted at interview:* “When our mobility slows down to a point where perhaps we can't drive, that might be just about the time [to move].” (P2)*

Most people either “loved” their community or found it “ok” to live there, although one participant stated he hated where he lived (see Table 2). Participants who drove everywhere by car were more likely to state that where they live is “ok” while most participants who used the car for 90–99% of all distance travelled stated that they loved where they lived. The main preferred aspects of the built environment by the participants included proximity, ambience, and access to public transport. Statements at interview related to these factors include: “*So it's all sort of within reach and it's nice.” (P10) *and* “You can get a bus anywhere over there.” (P9)*

However, most of the non-preferred features within their current environments were related to transportation (access to public transport, bikeability, and walkability). Safety was a main issue that deterred participants from using active transportation, with participants describing how limited sidewalks and the speed of pedestrian lights limited their mobility: *“People can't actually step off the road with safe access—on both sides of the road in some sections.” (P7)* and* “There are lights on the corner with a pedestrian crossing. I tried to get across as fast as I can and I can't get across in one change of the lights.” (P8)* Brisbane's hilly environment and the lack of well-maintained footpaths or bikeways were also perceived as key barriers to active commuting within the community. Participants explained how “*I would do a lot more walking if I could walk uphill and downhill.” (P6), “To get on the bikeways, you've still got to ride on roads.” (P3)* and* “You might see some scrapings along on the footpath and people could trip up.” (P13)*

Access to public transport was a topic raised mainly by people who drove the majority (90–100%) of their total travel distance. Some of these participants reported having good public transport services within their environment, describing how “*We are quite fortunate in that the bus goes past us either way, almost on a half-hourly basis.” (P2)* Within the same group, however, there were also participants who stated that public transport was not accessible where they lived:“*You can see why I push for a bus three/four times a week. There will come a time where I cannot drive anymore. How do I get to the shopping centre?” (P5)*

While affordability was one reason participants gave for living in their current environment, across all groups, proximity (to services, the city, friends and family) was the main reason given by older residents who drove for the majority (more than 90%) of the distance that they travelled during the monitored week.

### 3.3. Modes of Transport Used and Distance Travelled for out-of-Home Activities

The transportation system and built environmental features also had an impact on travel behaviour (see Table 3). Most participants stated that they would use the car, walk, and take the bus. Only a small number within the sample ever used a bicycle, train, or ferry. However, about half of participants reported that they would like to take public transport but that it was either too far from home or would not take them where they wished to go (or both in the case of one participant, see Table 3). One person identified busses as being overcrowded and services being too infrequent.

While the questionnaire data suggested that people use different forms of transport, the GPS tracking over the monitored week shows that the car was used for the majority of distance travelled by all but two participants. These two people did not drive at all, while the other participants travelled by car for between 75% and 100% of all distance between home and their various destinations. Participants, for whom the car accounted for 100% of the distance travelled over seven days, had travelled on average 27.3 kilometres further than the rest of the retired participants.

Engagement in active transportation modes such as walking and biking varied widely within the sample. Eight people engaged in active transportation. Five of them walked 1–4% of their total distances, one walked 5% and biked 8% of the total distance tracked, one walked everywhere and another one was working part-time and combined train (60% of kilometres travelled) and bike (40% of kilometres travelled), mostly for work-related travel. This particular participant travelled by bike as many kilometres as other participants did by car and overall, travelled the second greatest distance of the entire sample. The participant who walked 100% stated that he would usually drive a car himself but could not drive temporarily due to health issues. 

The GPS data showed that only five of the thirteen participants used public transport and that the extent of usage varied. Distance to CBD and frequency of services seem to influence its usage. Public transport was used by three participants, living 9 to 10 kilometres away from CBD, for 5% to 6% of the distance travelled. Only one of them had a quarter-hourly bus service available. Another participant, with a quarter-hourly bus service available, lived closer to the CBD and was using public transport for up to 18% of her travel. 

Trips were generally made between 7 am and 9 pm. Only four people engaged in recreational walking or cycling activities during the tracked week. 

## 4. Discussion

This research provides some significant insights into the impact of the suburban environment on the use of different transport options in older age. The qualitative study has provided a snapshot of travel behaviour of thirteen older people, gained by a combination of qualitative interviews, travel diaries, and unique person-based GPS observation of a week's travel. Combined, the findings highlight how suburban environments in Brisbane influence older people's travel behaviour.

Three key findings warrant specific attention. Firstly, low-density suburban environments are impractical for walking or biking for older people. Factors such as lack of footpaths or bikeways and the hilly environment create difficulties for older people wanting to use these forms of transportation. Consistent with previous research (e.g., [12]), our findings also suggest that suburban designs discourage older people from incorporating physical activity into their daily lives. This raises two critical aspects in relation to active ageing: the importance for physical activity in older age for health and the engagement in social encounters within the community [12]. People who walk or bike might be more engaged in their community, because the act of walking within the local community helps to foster an appreciation of it [10, 12]. This finding lends support for Alley et al. [10] proposition that the creation of safe walking and biking environments has the capacity to enhance quality of life in older age not only by encouraging physical activity but by also facilitating engagement with and appreciation of one's local community.

Secondly, low-density suburban environments often tend to make the use of public transportation impractical. Our findings show that most participants (across eleven suburbs) had access to public transport within a walking time of 5 to 7 minutes, but only a small number used public transport. Even though the distance between home and bus stops might be walkable, the quality of the pedestrian infrastructure might be discouraging older people from using public transport [14]. If the street environment does not support walking to and from public transport, it is likely to impede its use by older people. Infrequent or low frequency scheduling might also be an impact factor on the non-use by older people [14]. Our findings suggest that policy makers and transportation planners promoting active ageing might want to increase the use of public transportation by older people. This is critical as public transportation allows car-independent transportation within the wider community. Strategies need to be developed to encourage the use of public transportation by provision of a reasonable frequency of transportation throughout the day and a pedestrian friendly infrastructure at all stages of the journey between home, transit nodes and destinations as well. 

Thirdly, this research highlights that low-density environments are likely to create car dependency in older age. This is not a problem as long as the older people can drive. But, the car is not a sustainable transport option [32]—as our older participants themselves acknowledged. However, it is unlikely that older people begin to use public transport when they retire, especially if they never used public transport before [33]. The results of this study show that suburban environments put ageing in place at risk if driving is not longer an option. Interestingly, Lord et al. [26] found that older people change their lifestyle in order to remain in their communities as they age. Consequently, this adaption process also results in a change of travel behaviour and the reduction and fragmentation of the action space within the community [34]. This has implications for active ageing, as the reduced mobility could lead to reduced participation within the community. Retrofitting neighbourhoods is, in combination with a range of policy and program development, a possible way to reduce mobility lost for older people who are ageing in place [33]. While on one hand the aim should be to make suburban environments less car-dependent, it also would mean keeping older people driving safely as long as possible. 

Finally, this is a qualitative study with a small sample size conducted in Brisbane, Australia, which necessarily precludes its findings being generalised to older people living in other suburban contexts. It needs also to be acknowledged that the combination of Brisbane's subtropical climate and its hilly topography might make the use of active and public transportation more difficult in certain locations and at certain times of the year (e.g., the humidity and storms at heat of summer). The way GPS data was prepared and analysed in this research is likely to be infeasible for use among large samples. Still, the data collection from different sources allowed the researchers to capture the effect suburban environments can have on the use of transport options in older age. Given the nature of the findings, they are also likely to be relevant to suburban environments elsewhere. Of course, much more research (with larger, more diverse samples in different contexts) is needed to better understand the design characteristics of “age-friendly” environments and the relationship between mobility and activity within the suburban environment. 

## 5. Conclusion

Active ageing is a concept that encompasses preventive health, social participation, and overall wellbeing during the ageing process. Our findings suggest that environments that support active transportation modes not only allow older people opportunities for maximising their physical activity but also their use of public transportation and, in turn, their engagement within the wider community. Governments need to prioritise forms of urban development that create the conditions whereby older people are able to walk and bike safely and gain easy access to public transport. However, as older people might potentially be physically able to drive a car longer than they are able to use active and public transportation, the environment also needs to facilitate safe transportation by car in older age. Given that Australia's population is ageing, and that out-of-home mobility is critical to active ageing within the community, there is an urgent need for further attention to be paid to the impact of built environment characteristics and available transport options to encourage older people's mobility. This is critical for policymakers and planners to be better informed about the development of tailored strategies that will help ensure that people can remain active and engaged within the community as they age.

## Figures and Tables

**Table 1:** Demographic characteristics, residential character, and location.

	Participant car usage (percentage of distance travelled)												
	100%					90–99%				75–77%		0%	
	P1	P2	P3	P4	P5	P6	P7	P8	P9	P10	P11	P12	P13
Demographics													

Age	65	71	75	80	84	63	63	80	87	57	72	67	69
Gender													
Male		x	x	x	x			x			x	x	x
Female	x					x	x		x	x			
Marital status													
Married		x	x	x	x	x	x	x					
Widowed									x				
Not married										x	x		
Living alone	x												x
Living with friends/other people												x	
Paid work													
None	x	x	x	x		x	x	x	x	x	x		x
Part-time												x	
Annual income													
Under $20k					x				x	x		x	
$20k–$40k	x	x		x									x
$40k–$50k						x	x						
≥$70K–$100k			x					x			x		

Residential character and location													

Approx distance to CBD (in kms)	17.0	19.0	8.0	20.0	15.0	4.0	9.0	6.0	10.0	9.0	6.0	20.0	4.0
Density (people per hectare)	13.7	5.1	21.1	7.1	7.8	25.7	18.2	27.7	13.9	21.4	27.7	17.3	25.7
Available transport													
Car	x	x	x	x	x	x	x	x			x		x
Bus	x	x	x		x	x	x	x	x	x	x	x	x
Service frequency													
Quarter-hourly			x					x	x		x		
Half-hourly						x	x		x			x	x
Hourly	x	x	x		x	x	x		x	x		x	x
Peak time more frequent	x		x			x			x	x		x	x
Suitability of location for ageing in place													
Would need to relocate		x	x	x			x	x					
Could stay with help from family					x				x				
Could stay by changing current transport mode	x					x						x	x
Could stay by using local services	x												
Not thought about										x			

**Table 2:** Preferred and Non-preferred features of built environment.

	Participant car usage (percentage of distance travelled)												
	100%					90–99%				75–77%		0%	
	P1	P2	P3	P4	P5	P6	P7	P8	P9	P10	P11	P12	P13
Reasons for living in the community													
Affordability		x	x	x		x		x		x		x	
Proximity	x			x	x	x	x	x	x				
Liking		x	x				x	x	x				x
Safety		x					x	x	x				
Hometown											x		
Community member			x						x				
Staying in community													
As long as I am independent	x	x		x	x		x	x		x	x		
As long as I live						x			x				x
Until a set timeframe			x										
Liking of community													
Love it					x	x		x	x	x			x
It is ok	x	x	x	x			x				x		
Hate it												x	
Preferred features of built environment													
Proximity			x			x		x		x	x		x
Ambience	x				x		x				x		x
Wide streets		x											
Access to public transport		x	x			x			x				
Walkability		x						x					
Non-preferred features in built environment													
Lack of public green space							x						
Density			x	x				x		x			
Bikeability			x							x		x	
Walkability	x					x	x	x					x
Access public transport	x			x	x		x	x					
Information public transport													x
Crowded public transport													x
Design public transport				x									

**Table 3:** Travel behaviour and daily average distance travelled by mode of transport.

	Participant car usage (percentage of distance travelled)												
	100%					90–99%				75–77%		0%	
	P1	P2	P3	P4	P5	P6	P7	P8	P9	P10	P11	P12	P13
Travel behaviour													

Active transport													
Walk		x	x	x		x		x	x	x	x		x
Bike			x							x			
Car													
Drive myself	x	x	x	x	x	x	x	x			x		x
Someone else drives	x			x				x	x				
Public Transport													
Bus	x		x	x		x	x		x	x	x	x	
Train	x	x											
Ferry								x					
Reasons not to use public transport													
Unavailability of routes to preferred destinations	x						x	x					
Transit depot too far from home			x	x	x		x						
Overcrowded and infrequent													x

Daily average distance travelled per mode of transport (kilometres)													

Active transport													
Walk						0.1	0.2	0.3	0.2	0.6	0.2		6.3
Bike										1.0		24.8	
Car													
Drove myself	35.4	33.7	66.8	29.4	16.1	24.1	18.4	6,8			9.5		
Someone else drove	1.9	5.2		1.0				1.0	13.6	8.7			
Unspecified		2.2		3.3									
Public transport													
Bus							1.2		0.8	0.8	0.8		
Train												36.3	
Ferry											1.2	0.1	
Taxi											1.0		
Recreational													
Walk		0.4	0.1							1.8	3.7		
Bike			2.8										
